# Characterization of a newly identified ETV6-NTRK3 fusion transcript in acute myeloid leukemia

**DOI:** 10.1186/1746-1596-6-19

**Published:** 2011-03-15

**Authors:** Johanna M Kralik, Wolfgang Kranewitter, Hans Boesmueller, Renate Marschon, Gertraud Tschurtschenthaler, Holger Rumpold, Kurt Wiesinger, Martin Erdel, Andreas L Petzer, Gerald Webersinke

**Affiliations:** 1 Laboratory of Molecular Biology and Tumorcytogenetics, Department of Internal Medicine I, Krankenhaus der Barmherzigen Schwestern, Seilerstaette 4, 4010 Linz, Austria; 2 Institute of Pathology, Krankenhaus der Barmherzigen Schwestern, Seilerstaette 4, 4010 Linz, Austria; 3 Laboratory for Hematology, Department of Internal Medicine I, Krankenhaus der Barmherzigen Schwestern, Seilerstaette 4, 4010 Linz, Austria; 4 Department of Internal Medicine I; Krankenhaus der Barmherzigen Schwestern, Seilerstaette 4, 4010 Linz, Austria; 5 Institute of Laboratory Medicine BB&BS, Krankenhaus der Barmherzigen Schwestern, Seilerstaette 4, 4010 Linz, Austria

## Abstract

**Background:**

Characterization of novel fusion genes in acute leukemia is important for gaining information about leukemia genesis. We describe the characterization of a new *ETV6 *fusion gene in acute myeloid leukemia (AML) FAB M0 as a result of an uncommon translocation involving chromosomes 12 and 15.

**Methods:**

The *ETV6 *locus at 12p13 was shown to be translocated and to constitute the 5' end of the fusion product by *ETV6 *break apart fluorescence in situ hybridisation (FISH). To identify a fusion partner 3' rapid amplification of cDNA-ends with polymerase chain reaction (RACE PCR) was performed followed by cloning and sequencing.

**Results:**

The *NTRK3 *gene on chromosome 15 was found to constitute the 3' end of the fusion gene and the underlying *ETV6-NTRK3 *rearrangement was verified by reverse transcriptase PCR. No RNA of the reciprocal *NTRK3*-*ETV6 *fusion gene could be detected.

**Conclusion:**

We have characterized a novel *ETV6-NTRK3 *fusion transcript which has not been previously described in AML FAB M0 by FISH and RACE PCR. *ETV6-NTRK3 *rearrangements have been described in secretory breast carcinoma and congenital fibrosarcoma.

## Background

Chromosomal translocations resulting in fusion genes with transforming activity are fundamental in leukemia genesis. The *ETV6 *gene (*ETS *variant gene 6) on the short arm of chromosome 12 encodes a transcriptional repressor of the *ETS *transcription factor family which is fundamental in adult hematopoiesis and plays a versatile role in hematological malignancies [1]. *ETS *family members are essential for a variety of cellular processes including proliferation, differentiation, migration and tissue remodeling, angiogenesis, apoptosis, as well as hematopoiesis and cell transformation [2,3]. ETV6 is a 452 amino acid protein encoded by 8 exons containing the characteristic ETS domain at its carboxy terminus [4,5]. The ETS domain harbors DNA binding properties but also mediates protein-protein interactions [1]. ETV6 contains a helix-loop-helix (HLH) domain at its amino terminus which mediates homotypic oligomerization of ETV6 molecules and also heterotypic interactions with other proteins [1,2]. *ETV6 *is located on band 12p13 which is genetically unstable and thus susceptible to chromosomal rearrangements [6]. In recent years *ETV6 *was shown to be involved in a variety of translocations associated with hematological malignancies of both myeloid as well as lymphoid origin [1,7]. The diversity of *ETV6 *rearrangements is striking considering that the breakpoints do not cluster within a particular region of the gene [4].

Fusions between *ETV6 *and phospho-tyrosinkinases (*PTK*s) are found in a variety of hematological diseases where the HLH domain is essential for the aberrant function of the fusion product, e.g. chronic myelomonocytic leukemia (CMML), AML, acute lymphoid leukemia (ALL) or myelodysplastic syndrome (MDS) [1]. In other cases ETV6 has been shown to contribute to the dysregulation of cellular functions by its ETS domain [8].

In the present study we identified the PTK NTRK3 as an ETV6 fusion partner. NTRK3 is a membrane anchored tyrosine kinase and functions as a receptor for the neurotrophin NT-3, regulating development and maintenance of the vertebrate nervous system [9,10]. NTRK3 does not only show expression in neural tissues but also in hematopoietic and epithelial cells [10].

The *NTRK3 *gene is composed of 18 exons; the receptor kinase domain is encoded by exons 13 through 18. Alternative splicing of *NTRK3 *leads to different isoforms, one of which is a truncated version of the full-length protein and does not possess a catalytically active kinase domain but exhibits exons 13b and 14b as its final two exons [9]. The catalytically active ETV6-NTRK3 fusion proteins have been previously detected in non-hematopoietic tumors, e.g. secretory breast carcinoma and congenital fibrosarcoma and in only one single case of hematological malignancy, i.e. an AML [11-13].

## Methods

### Patient profile

The 55-year-old male patient was transferred to our department with a newly diagnosed AML-M0 according to the FAB-classification. The bone marrow showed a 45% infiltration with AML-blasts, which stained positive for CD33, CD13, CD56 and CD7. Due to that, the patient received a treatment consisting of one induction of cytarabine and idarubicin followed by a second induction of cytarabine and mitoxantrone, a salvage therapy consisting of cytarabine and fludarabine followed by two cycles of gemtuzumab ozogamicine. The leukemic clone was refractory after each treatment cycle with an unchanged blast count in the bone marrow and the patient subsequently died after a short period of best supportive care.

### Molecular and cytogenetic diagnostics

The patient was tested negative for mutations of the *NPM1 *and *FLT3 *genes and *AML1-ETO*- and *CBFb-MYH11 *rearrangements.

**Figure 1:**
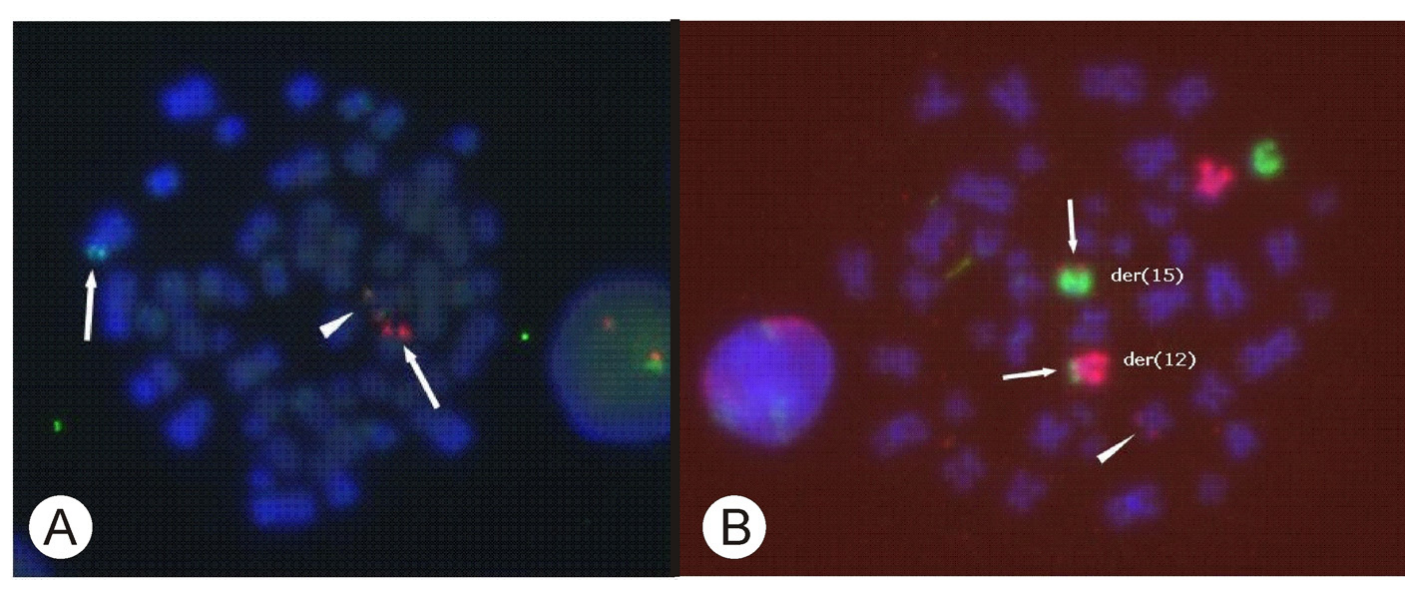
**FISH experiments**. 1a. *ETV6 *break apart FISH. Arrows indicate the split signal for one allele, the arrow head marks the fusion signal of the wildtype allele. 1b. WCP of chromosomes 12 (red) and 15 (green). The arrows indicate the reciprocal translocation giving rise to derivate chromosomes der(12) and der(15). The arrow head marks the additional material of chromosome 12 on chromosome 10.

Conventional karyotyping using GTG banding of unstimulated bone marrow cultures showed a pathological cell line exhibiting monosomy 7 and a translocation t(10;12)(q24;p13). An *ETV6 *rearrangement was assumed and could be demonstrated by Dual Color Break Apart FISH (LSI^® ^ETV6 probe; Abbott Molecular) (Figure 1A). Whole chromosome paints (WCPs) (XCP Human Chromosomen Paints; Metasystems) revealed additional material of chromosome 12 on a second chromosome due to a cryptic translocation; chromosome 15 could be identified as a partner. (Figure 1B)

### RACE PCR method

3' nested RACE PCR was performed with the GeneRacerTM Kit (Invitrogen) according to the manufacturer protocol. Primers ETV6A/nested, ETV6B/nested and ETV6C/nested were used (ETV6 GenBank Accession number NM_001987, Version NM_001987.4, see Additional file 1 Table S1 for sequence information) with Platinum^® ^*Taq *DNA Polymerase High Fidelity (Invitrogen; 5 U/μl).

Nested RACE PCR products of interest were excised from the gel and cloned using the TOPO TA Cloning^® ^Kit (Invitrogen) according to the manufacturer's recommendations.

Amplification of cloned RACE PCR products was performed with 0,5 - 1,0 ng plasmid DNA, M13F/R primers and AmpliTaq Gold™DNA Polymerase (5 U/μl; Applied Biosystems). PCR products were purified using the ExoSAP-IT^® ^system (USB) and subsequently sequenced using the BigDye^® ^Terminator v1.1 Cycle Sequencing Kit (Applied Biosystems). Capillary electrophoresis was run in the ABI 3130 Genetic Analyzer (Applied Biosystems).

### Reverse transcriptase PCR for expression testing

cDNA was prepared from total RNA by random hexamer priming (Amersham Biosciences) using M-MLV RT as indicated by the manufacturer (Promega).

For reverse-transcriptase PCR reactions cDNA was used undiluted with specifically designed primers for *ETV6 *(ETV6 GenBank Accession number NM_001987, Version NM_001987.4) and *NTRK3 *exons (GenBank Accession number NM_001012338, version NM_001012338.1 and GenBank Accession number NM_001007156, version NM_001007156.1) as listed in the Additional file 1 Table S1.

## Results and Discussion

Genetic alterations play a key role in the pathogenesis of both solid tumors and hematological malignancies. In recent years the description of these alterations by molecular genetics provided crucial information for risk stratification and therapy, especially for acute leukemias. We describe a novel *ETV6 *fusion in an AML patient, which was confirmed by FISH and WCP (Figure 1) and could not be detected by routine chromosome banding techniques because it is highly cryptic. Therefore, the translocation is easily overlooked in conventional cytogenetics.

By RACE PCR and sequencing a truncated version of the *NTRK3 *transcript was found in the forward sequencing reaction containing the polyA tail of the transcript, whereas the reverse reaction identified *ETV6 *as the 5' part of the transcript. *NTRK3 *is located on chromosome 15q25 which is affected in our patient as demonstrated by WCP 12 FISH (Figure 1). Out of 142 sequencing reactions of RACE products, no partner gene on chromosome 10 could be detected. Whole chromosome paints of chromosomes 10 and 15 did not show any translocated signals (data not shown).

**Figure 2:**
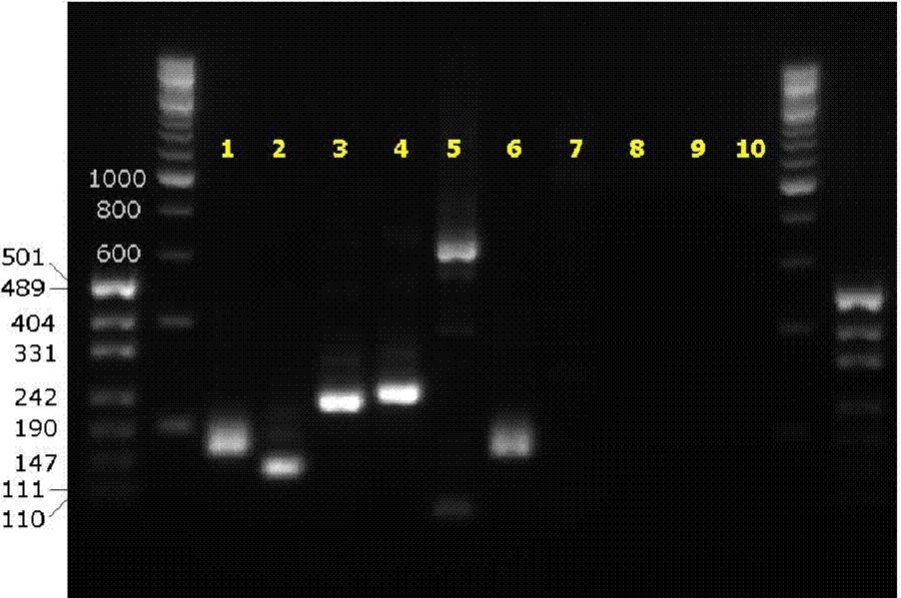
**Nested RT-PCR of fusion transcripts**. Results indicate that *ETV6 *builds fusion transcripts with both the truncated and catalytically active *NTRK3 *(lane numbers explained below). *ETV6-NTRK3 *rearrangement: 1 control PCR of patient with *ETV6-NTRK3*-rearrangement (*ABL1*); 2 *ETV6 *exon5 and *NTRK3 *exon14; 3 *ETV6 *exon5 and *NTRK3 *exon14I; 4 *ETV6 *exon5 and *NTRK3 *exon14II; 5 *ETV6 *exon5 and *NTRK3 *exon17; all *NTRK3 *primers are specific for the tyrosine kinase domain. Controls: 6 control PCR of patient without *ETV6-NTRK3*-rearrangement (*ABL1*); 7 *ETV6 *exon5 and *NTRK3*SequF (patient without *ETV6-NTRK3*-rearrangement); 10 water control. *NTRK3-ETV6 *Rearrangement: 8,9 *NTRK3 *exon12 (2 primers) and *ETV6 *exon6.

**Figure 3:**
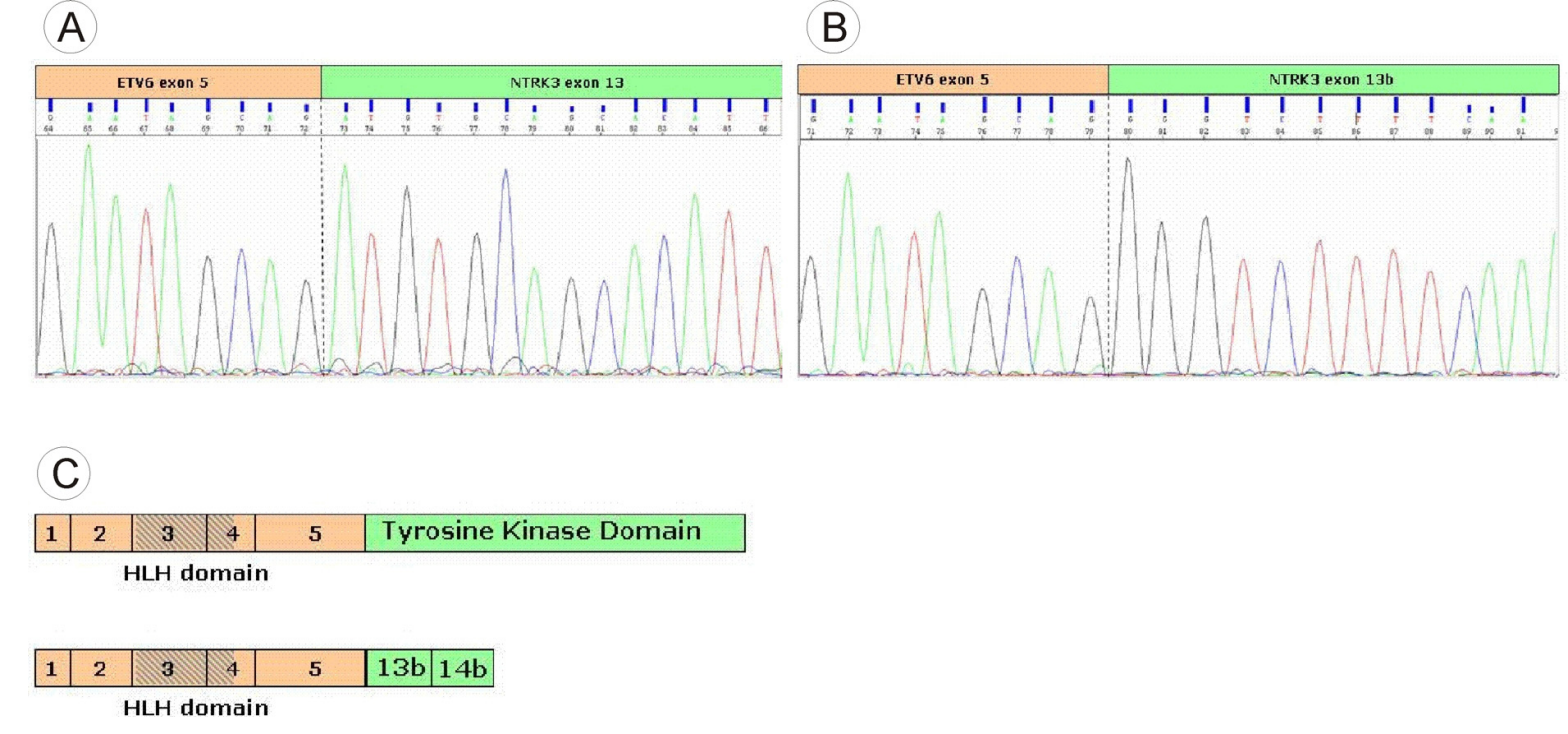
**Sequences of fusion transcripts**. Electropherogram showing the fusion transcripts involving *ETV6 *and the catalytically active *NTRK3 *isoform (3a) and *ETV6 *fused to the truncated *NTRK3 *isoform (3b). In either case, sequencing was done in both directions with only one being shown. (3c) Proposed structure of the entire fusion transcripts. *ETV6 *(pale orange) being fused to the catalytically active *NTRK3 *(green) isoform (upper) and to the truncated isoform of *NTRK3 *(lower), resp.

Expression of the truncated fusion transcript and possible isoforms was tested in nested RT-PCR experiments (see Figure 2). Results showed that *ETV6 *and the alternatively spliced, truncated *NTRK3 *clearly exist as a fusion transcript. Furthermore, the existence of *ETV6 *being fused to catalytically active *NTRK3 *was proven. RT-PCR amplification products of patient RNA were then sequenced with appropriate primers. Based on these results the proposed entire fusion transcripts contain *ETV6 *exons 1 through 5 fused to *NTRK3 *exons 13b and 14b or *NTRK3 *exons 13 through 18 (see Figure 3). Both *NTRK3 *isoforms are in-frame fusion partners of *ETV6*. No *ETV6-NTRK3 *transcripts could be detected in control cDNA from patients without *ETV6 *rearrangement. The reciprocal translocation product (*NTRK3-ETV6*) was not detected on the RNA level when patient cDNA was amplified with different primer combinations suggesting its missing expression.

So far, catalytically active ETV6-NTRK3 fusion proteins have been detected in a significant fraction of non-hematopoietic tumors, e.g. secretory breast carcinoma, congenital fibrosarcoma or mesoblastic nephroma [11,12,14]. Their transforming ability is based on constitutive activation of the NTRK3 moiety with downstream activation of Ras- MAP kinase and phosphatidyl inositol-3-kinase (PI3K)-AKT pathways [11]. Interestingly, the *ETV6-NTRK3 *fusion has been reported in only one case of hematological malignancy, i.e. an AML (FAB M2) [13]. Here, we describe the presence of the *ETV6-NTRK3 *fusion in a further AML case which, in contrast belongs to the FAB M0 subgroup. Furthermore, there are two striking differences between the previously detected hematological *ETV6-NTRK3 *fusion and the one described here. In this context, one needs to point out that ETV6 activity as a transcriptional repressor is achieved by at least two different domains: the HLH domain and the central region located between the HLH and ETS domain encoded by part of exon 4 and the entire exon 5 [1,2]. This central domain was shown to interact with corepressors, whereas the HLH domain represses gene transcription independently from corepressors [2,15]. As far as the differences are concerned, the fusion transcript identified in this study exhibits *ETV6 *exons 1 through 5 in contrast to the previously published case which contained only the first four *ETV6 *exons. Therefore, the entire central domain, made up partly of exon 4 and the entire exon 5, is still present and might enable further protein-protein interactions. The second difference is that *ETV6 *was shown to be fused to the catalytically active isoform in the earlier report. In our case we demonstrated that alternative splicing of the transcribed *ETV6-NTRK3 *gene takes place and gives rise to the truncated NTRK3 isoform which is known to induce signaling that results in membrane ruffling and cellular protrusions, structural changes that support cell migration [16].

Unfortunately, insufficient patient material did not allow the investigation of ETV6-NTRK3 expression on the protein level. The oncogenic activity of both variants remains to be revealed. Thereby one must keep in mind that fusion products containing ETV6 have been shown to involve diversely regulated aberrant mechanisms. As far as kinase domain proteins are concerned, ETV6 influences the fusion partner's activity via its HLH (dimerization) domain [8]. Regarding the ETV6-NTRK3 fusion, data prove that the NTRK3 kinase domain by itself does not provoke transformation activity. More precisely, it was demonstrated that NIH3T3 cells cannot be successfully transformed by ETV6-NTRK3 chimeric proteins that still possess a perfectly operating NTRK3 kinase domain but lack a functional ETV6 HLH domain [17]. In other rearrangements, the *ETV6 *promotor driving the transcription of the fusion gene is thought to confer oncogenic potential [8]. It is possible that the two variants of fusion products identified in this study may function differently in cell transformation.

## Conclusion

We described a novel *ETV6 *fusion with a gene involved in neuronal development, *NTRK3*, in AML. The fusion gene has been previously detected in non-hematopoietic tumors, e.g. secretory breast carcinoma and congenital fibrosarcoma. Comparable to other molecular alterations in AML the *ETV6-NTRK3 *fusion might be of prognostic relevance and should be investigated further in larger patient cohorts.

## Competing interests

The authors declare that they have no competing interests.

## Authors' contributions

KJM designed and performed research, analyzed data and wrote the paper, KW and MR contributed to molecular studies, TG performed FACS analysis, RH and PA contributed clinical data, WK did bone marrow cytology, BHC did bone marrow histology, EM contributed to cytogenetics, WG designed the study, did cytogenetics, analysed the data and cowrote the paper. All authors read and approved the final manuscript.

## Supplementary Material

Additional file 1 **Table S1**. Primer sequences used in RT-PCR and sequencing analyses. Click here for file
