# Supplementary material for: Characterization of a newly identified ETV6-NTRK3 fusion transcript in acute myeloid leukemia
Source: Diagn Pathol. 2011 Mar 15;6:19. doi: 10.1186/1746-1596-6-19 (PMC3063188; doi:10.1186/1746-1596-6-19)
Supplement: Additional file 1 — Table S1. Primer sequences used in RT-PCR and sequencing analyses. [file 1746-1596-6-19-S1.DOC]

**Additional file 1, Table S1:**

| Designation | Sequence (5’ 🡪 3’) |
| --- | --- |
| ABL1 control a | GCC TCA GGG TCT GAG TGA AGC CGC TCG TTG |
| ABL1 control b | TGT GAT TAT AGC CTA AGA CCC GGA GCT TTT C |
| ETV6A | CAT ATA CAC CTC CAG AGA GCC CAG TGC |
| ETV6Anested | CTT CAT GTT CCA GTG CCT CGA GCG C |
| ETV6B | CTG AAG CAG AGG AAA CCT CGG ATT C |
| ETV6Bnested | CAC CCT GGA AAC TCT ATA CAC ACA CAG C |
| ETV6C | CAT GCC CAT TGG GAG AAT AGC AGA CTG |
| ETV6Cnested | CTG ACA GCC GGT ACG AAA ACT TCA TCC |
| ETV6 exon 6 | GAT TCT TTG TCC TCC CAT CG |
| ETV6 exon 5 | ATG CCC ATT GGG AGA ATA GC |
| ETV6 Sequ a | CCC ATC AAC CTC TCT CAT CG |
| ETV6 Sequ b | CTC TCT CAT CGG GAA GAC C |
| M13F | TGT AAA ACG ACG GCC AGT |
| M13R | CAG GAA ACA GCT ATG ACC |
| NTRK3 exon12 a | GGA CAC AAC TGC CAC AAG C |
| NTRK3 exon12 b | CAT CCC TGT CAT TGA GAA CC |
| NTRK3 exon14 a | AAC TTG ACA ATG TGC TCA TGC |
| NTRK3 exon14 b | CCG CAC ACT CCA TAG AAC |
| NTRK3 exon17 | GAA CTT CCG GTA CAT GAT GC |
| NTRK3 Sequ | AAG ACA CTT CCC CAC TCT GG |
